# Genetic structure of sigmodontine rodents (Cricetidae) along an altitudinal gradient of the Atlantic Rain Forest in southern Brazil

**DOI:** 10.1590/S1415-47572009005000081

**Published:** 2009-12-01

**Authors:** Gislene L. Gonçalves, Jorge R. Marinho, Thales R. O. Freitas

**Affiliations:** 1 Departamento de Genética, Instituto de Biociências, Universidade Federal do Rio Grande do Sul, Porto Alegre, RSBrazil; 2 Departamento de Zoologia, Universidade Federal do Rio Grande do Sul, Porto Alegre, RSBrazil

**Keywords:** *Euryoryzomys russatus*, gene flow, mismatch distribution, *Oligoryzomys nigripes*, population expansion

## Abstract

The population genetic structure of two sympatric species of sigmodontine rodents (*Oligoryzomys nigripes* and *Euryoryzomys russatus*) was examined for mitochondrial DNA (mtDNA) sequence haplotypes of the control region. Samples were taken from three localities in the Atlantic Rain Forest in southern Brazil, along an altitudinal gradient with different types of habitat. In both species there was no genetic structure throughout their distribution, although levels of genetic variability and gene flow were high.

The genetic structure of populations is a necessary and important task for better understanding the history and future evolutionary potential of a species and its populations, especially from a conservation perspective (Burgman *et al.* 1993; Patton *et al.* 1996). The rodent subfamily Sigmodontinae comprises about 371 species, grouped into eight tribes (Wilson and Reeder 2005). Oryzomyini is a specious assemblage (Reig 1984, 1986) that encompasses 15 genera, including *Euryoryzomys* Weksler *et al.*, 2006 and *Oligoryzomys* Bangs, 1900, the latter being first proposed as a subgenus of *Euryoryzomys*. Further reviews based on morphological (Carleton and Musser 1989) and molecular data (Dickerman and Yates 1995; Myers *et al.* 1995; Weksler 2003) have supported the monophyly of *Oligoryzomys*.

*Oligoryzomys nigripes* (Olfers, 1818) is a small mouse (averaging 25 g in body mass) that occurs in grasslands and forests in Brazil (Mares *et al.* 1989; Stallings 1989; Vieira and Marinho-Filho 1998), and is considered a habitat-generalist species (Dalmagro and Viera 2005). It is characterized by the tail being longer than the head and body together, short and broad hind feet, a small skull, and a relatively broad, stocky rostrum. On the other hand, *Euryoryzomys russatus* (Wagner, 1848) is a terrestrial rodent, typically found in forest areas, with a medium-sized body (averaging 60 g in body mass) (Marinho 2004). Both species feed on seeds, fruits, and insects (Emmons and Feer 1990; Powers *et al.* 1999). These two species were selected for studying due to differences in both life history and habitat range, features that are likely to influence their respective genetic population structures. Furthermore, they are poorly known from an ecological perspective, only a few population studies having been reported so far (Chiappero *et al.* 1997; Perini *et al.* 2004; Trott *et al.* 2007). In this study, we investigated the fine-scale genetic structure of these two sympatric species of rodents, sampled from the same set of localities along a 58 km altitudinal gradient with different types of habitat. The sampling area consisted of three localities along an altitudinal gradient (30, 350, and 780 m) in the Atlantic Rain Forest, southern Brazil (Figure 1). The predominant types of habitat consisted of two major classes of vegetation according to the IBGE (1986): Dense Ombrophilous Forest (DOF) and Mixed Ombrophilous Forest (MOF). The DOF is subdivided into minor classes: Lowland Swamp Forest (LSF) which occurs from sea level up to 30 m a.s.l., Montane Forest, from 30 to 400 m a.s.l., and Sub-Montane Forest, over 400 m a.s.l. The two latter subdivisions will be considered as DOF *sensu stricto*. All the individuals (*O. nigripes*, n = 55; *E. russatus*, n = 30) were captured with live traps. DNA was extracted from frozen liver samples according to a protocol described by Medrano *et al.* (1990). We amplified part of the control region (410 bp) of the mtDNA via the polymerase chain reaction (PCR). Amplification was performed using the forward primer LBE08 that aligns to the tRNAthr gene flanking the control region and the reverse primer H12S (Sullivan *et al.* 1995, Rodrigues-Serrano *et al.* 2006). PCR conditions were the same as those described by Smith and Patton (1993). PCR products were purified with shrimp alkaline phosphatase and exonuclease I (Invitrogen, Carlsbad, California) and sequenced by using an ABI PRISM 3100 (Applied Biosystems Inc., Foster City, California). Sequence electropherograms were aligned in CLUSTAL W (Thompson *et al.* 1997). Haplotype diversity (Hd; Nei 1987) and the mean number of pairwise differences (π; Tajima 1983) were estimated by using ARLEQUIN 3.1 (Schneider *et al.* 2000). Genetic differentiation between populations was characterized by estimating pairwise *F*_ST_ (Weir and Cockerham 1984) using the unique haplotype model from ARLEQUIN. Topological relationships between control region haplotypes were estimated using the median-joining approach (Bandelt *et al.* 1999) implemented in NETWORK 4.5 (Fluxus Technology Ltd, Suffolk, England).

Patterns of genetic variability in *Oligoryzomys nigripes* and *E. russatus* were similar (Table 1). Perini *et al.* (2004), when estimating genetic variability by means of electrophoresis data among populations and species of *Oligoryzomys* and *Oryzomys*, also found similar levels of diversity in both genera. The number of variable sites identified in these species can be considered moderately high when compared to other studies with mtDNA sequences (Myers *et al.* 1995; Palma *et al.* 2005), perhaps due to the rapid rate of evolution in the control region.

Overall gene-flow estimates yielded a low and nonsignificant value for *O. nigripes*, *F*_ST_ = 0.015 and *E. russatus*, *F*_ST_ = 0.013, thereby indicating the lack of genetic structure among populations. Myers *et al.* (1995) studied mtDNA sequences (cytochrome b) in several species of *Oligoryzomys*, and found very little evidence of differentiation among their populations. Similar results were obtained by Trott (2000) when using RAPD markers in populations of six species of *Oligoryzomys*, including *O. nigripes*. An enzyme-electrophoretic study by Chiappero *et al.* (1997) estimated gene flow among populations of *Oligoryzomysflavescens* from Argentina, and found a lack of isolation-by-distance pattern among these populations. The haplotype network topologies of *O. nigripes* and *E. russatus* are shown in Figure 2. These species are characterized by low nucleotide diversity and high haplotype diversity, suggesting that their populations are composed of a large number of closely related haplotypes.

**Figure 1:**
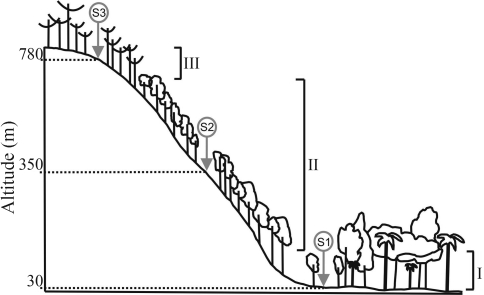
Atlantic Rain Forest profile showing distribution of sample sites (S) along an altitudinal gradient: S1), Terra de Areia Municipality; S2), Itati Municipality; S3), São Francisco de Paula Municipality. Plant cover: I, Lowland Swamp Forest; II, Dense Ombrophilous Forest; and III, Mixed Ombrophilous Forest.

**Figure 2:**
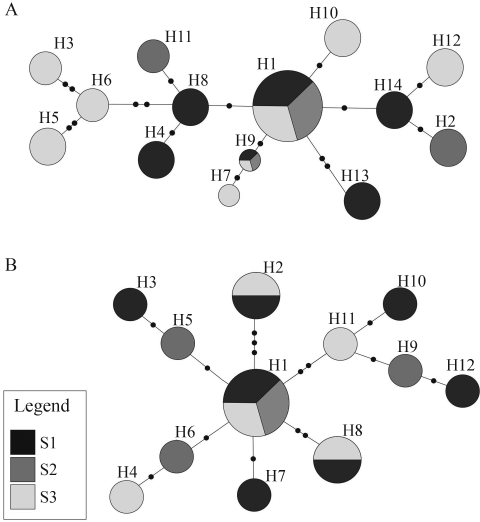
Median-joining network. Central haplotype, which are usually also the most common, are indicated by the larger circle. Each bar on the connections between haplotypes represents a unique mutational event (base substitution). Haplotypes are numbered. (A) *Oligoryzomys nigripes*; (B) *Euryoryzomys russatus*.

Both sigmodontine species exhibited no population genetic structuring, although they showed similar patterns of shared haplotypes within the different types of habitat and altitudes of the rainforest. According to Schoener (1974), similar species that coexist spatially generally show differences in feeding strategies, occupy different habitats, or have distinct temporal patterns of activity. On analyzing field data, it can be inferred that *O. nigripes* was more abundant in dense and mixed ombrophilous forest, whereas *O. russatus* was more so in lowland swamp forest (Marinho 2004). However, the lack of genetic structure, as found in this study, indicates that both sigmodontine species do not show specificity for the habitat types, a pattern different than that seen in other sigmodontine rodents, such as *Delomys dorsalis* (Cademartori *et al.* 2002) and *Akodon reigi* (Geise *et al.* 2004).

## Figures and Tables

**Table 1:** Measurements of genetic variability for each of the species examined, by geographical region (S1, Lowland Swamp Forest; S2, Dense Ombrophilous Forest; S3, Mixed Ombrophilous Forest): *n*, number of individuals; *nh*, number of haplotypes; *Hd*, haplotype diversity; and π, nucleotide diversity.

Species/locality	*n*	*nh*	*Hd*	π
*Oligoryzomys nigripes*				
S1	24	6	0.80	0.0174
S2	10	4	0.86	0.0101
S3	21	8	0.89	0.0048
Σ	55	14	0.85	0.0107

*Euryoryzomys russatus*				
S1	17	7	0.81	0.0045
S2	5	4	0.96	0.0055
S3	8	5	0.87	0.0047
Σ	30	12	0.88	0.0049
